# Changes in extreme temperature over China when global warming stabilized at 1.5 °C and 2.0 °C

**DOI:** 10.1038/s41598-019-50036-z

**Published:** 2019-10-18

**Authors:** Cenxiao Sun, Zhihong Jiang, Wei Li, Qiyao Hou, Laurent Li

**Affiliations:** 1 Joint International Research Laboratory of Climate and Environment Change, Collaborative Innovation Center on Forecast and Evaluation of Meteorological Disaster, Nanjing University of Information Science and Technology, Nanjing, China; 2 Key Laboratory of Meteorological Disaster of Ministry of Education, Collaborative Innovation Center on Forecast and Evaluation of Meteorological Disaster, Nanjing University of Information Science and Technology, Nanjing, China; 3 Laboratoire de Météorologie Dynamique, IPSL, CNRS, Sorbonne Université, Ecole Normale Supérieure, Ecole Polytechnique, Paris, France

**Keywords:** Atmospheric science, Climate change

## Abstract

The 1.5 °C global warming target proposed by the Paris Agreement has raised worldwide attention and inspired numerous studies to assess corresponding climate changes for different regions of the world. But CMIP5 models based on Representative Concentration Pathways (RCP) are ‘transient simulations’ and cannot reflect the response of climate warming stabilized at 1.5 °C. The current work presents an assessment of extreme temperature changes in China with simulations from ‘Half a degree Additional warming, Prognosis and Projected Impacts’ (HAPPI) project specially conceived for global warming levels stabilized at 1.5 °C and 2.0 °C. When global warming stabilizes at 1.5 °C/2.0 °C, the areal-mean temperature for whole China increases by about 0.94 °C/1.59 °C (relative to present period, taken from 2006–2015). Notable increase regions are mainly found in Northwest and Northeast-North China, but warm spell duration increases mostly in Southeast China. The effect of the additional 0.5 °C warming is particularly investigated and compared between the transient and stabilized simulations. Changes of mean and extreme temperature are larger in transient simulations than in stabilized simulations. The uncertainty range is also narrower in stabilized simulations. Under stabilized global warming scenario, extreme hot event with return period of 100 years in the present climate becomes event occurring every 4.79 (1.5 °C warming level) and 1.56 years (2.0 °C warming level), extreme cold event with return period of 10 years becomes event occurring every 67 years under 1.5 °C warming and is unlikely to occur under 2.0 °C warming. For geographic distribution, the occurrence probabilities of extreme (hot and cold) events mainly change in the Tibetan Plateau, and the extreme cold events also change in Northeast and Southeast China.

## Introduction

Global warming exacerbates the threat of climate change to ecology and human societies^1^. To combat climate change, the international community in December 2015 adopted the Paris Agreement that aims at holding the increase in the global average temperature to well below 2.0 °C above pre-industrial levels and pursuing efforts to limit the temperature increase to 1.5 °C^2^. The Global Warming of 1.5 °C report released in October 2018 points out that the global mean surface temperature has already increased by approximately 1.0 °C, and will reach 1.5 °C around 2030–2052 if the current warming rate continues^3^.

Researchers have conducted extensive researches focusing on the 1.5 °C warming target. However, these studies were mainly based on simulations under Representative Concentration Pathways (RCP) scenarios, for example, using datasets from global climate system models of the 5th phase of the Coupled Model Inter-comparison Project (CMIP5). RCP scenarios are devoted to projecting the transient response of climate to specific radiative forcing conditions^3^. Researchers usually choose a period centered at the year of 1.5 °C or 2.0 °C global warming above pre-industrial levels to analyze climate responses for the two specific warming targets. During this time the global mean surface temperature keeps increasing, due to the transient feature of the simulations. Results based on transient simulations show that mean temperature increases mostly in mid-high latitudes of the Northern Hemisphere under 1.5 °C global warming^4–7^. Compared to the change of mean temperature, response of extreme events to global warming varies from region to region^8–12^. Extreme high temperature increases in most parts of the Northern Hemisphere and southern Africa under 2.0 °C global warming, while the magnitude of increase reduces under 1.5 °C warmer world^10,13–15^. Limiting global warming at 1.5 °C can effectively reduce the population size affected by extreme climate compared to 2.0 °C global warming^16–18^.

However, results based on transient simulations may suffer uncertainties due to climate internal variability. We need to find a suitable way to make the global mean surface temperature stabilized at specific warming targets, i.e., 1.5 °C and 2 °C above pre-industrial levels^19,20^. Moreover, the small ensemble size of CMIP5 (a few members) constitutes another obstacle to suitably assess the uncertainty in relation to climate extremes^19,21^. To remediate these issues, the ‘Half a degree Additional warming, Prognosis and Projected Impacts’ (HAPPI) project proposed an alternative modelling strategy. An examination of all available CMIP5 simulations under RCP2.6 reveals that by chance the end of the 21^st^ century has the global warming level of 1.55 °C, compared to pre-industrial period (1861–1880). If we subtract the sea-surface temperature and sea-ice anomalous fields from those in RCP8.5 scenario present time (2006 to 2015, reference period), we can then superimpose such anomalous fields onto the observed sea-surface temperature and sea-ice fields from 2006 to 2015. For the 2 °C warming level, the end-of-the-century fields are a combination of weighted RCP2.6 and RCP4.5 to ensure the target warming. Actually the 1.5 °C global warming level (from pre-industrial 1861–1880) shows a global warming of about 0.7 °C from the reference period (2006–2015), and the 2.0 °C warming level shows a global warming of 1.2 °C. For the reference and the two perturbed levels, one can thus perform ‘stabilized simulations’ (of AMIP-type), and with a large ensemble size. The large ensemble size (≥100) in each model of the HAPPI project can help us to estimate the uncertainty caused by internal variability^22^, especially when dealing with extreme events^19^.

Since the release of HAPPI data, many interesting studies using HAPPI have been reported in literature for stabilized climate response in different regions. Several hot spots have been revealed in Asia in the 1.5 °C warmer world, while extreme high temperature increases from about 0.5 to 1.5 °C (relative to present time) over most land areas and the intensity of heat waves also strengthens^23–25^. Harrington *et al*.^26^ pointed out that compared to 1.5 °C warming, the risk of extreme high temperature in South Asia and East Africa increased evidently under 2.0 °C global warming. Lewis *et al*.^27^ indicated that the heat extremes in Australia may increase by twice the global-mean warming. However, few studies with HAPPI are reported for climate change in China. We want to fill up this gap with the present study, and we believe that East Asia provides a favorable test-bed to investigate behaviors of extreme temperature events in stabilized and transient simulations respectively, considering the unique geographic location and regional terrain.

The paper is organized as the following. The datasets and methods used are introduced in section 2. Section 3.1 describes the response of extreme temperature in China to 1.5 °C, 2.0 °C and the additional 0.5 °C global warming, and we also perform an analysis of variance for HAPPI multi-member simulations. In section 3.2 and 3.3, we analyze changes of occurrence probabilities for extreme temperature events under different warming targets. We also use CMIP5 experiments to present an analysis of temperature extremes over China under transient warming simulations, which contributes to assess the reliability of our research.

## Data and Methods

### Data

The HAPPI datasets that we downloaded are the 3 simulations of the Tier-1 experiment: (1) present-day climate (2006–2015); (2) 1.5 °C warming level (relative to pre-industrial) for which the actual warming is about 0.7 °C if present-day is taken as reference; (3) 2.0 °C warming level (relative to pre-industrial) for which the actual warming is about 1.2 °C compared to present-day. The detailed experimental protocol can be found in Mitchell *et al*.^19^. Our analysis is performed on daily mean, minimum and maximum surface air temperature from CanAM4, ECHAM6-3-LR, MIROC5 and NorESM1-HAPPI models contributing to HAPPI (as shown in Table 1). Relevant diagnoses, including calculation of extreme indices, are performed on their native grids to achieve the highest accuracy possible. But to facilitate operations such as data visualization or areal average, indices calculated from different models with different resolutions are re-gridded to a common 1° × 1° grid with a bilinear interpolation scheme.

**Table 1:** Basic information on 4 HAPPI models used in this study.

Model	Resolution (#lat × #long)	Ensemble members
CanAM4	64 × 128	100
ECHAM6-3-LR	96 × 192	100
MIROC5	128 × 256	100
NorESM1	192 × 288	100

In order to assess eventual differences between transient and stabilized simulations for the half-a-degree additional warming, we also use datasets under RCP4.5 scenario from 15 models in CMIP5 (as shown in Table 2). A single member (r1i1p1) is used for each model, and the 1.5 °C/2.0 °C global warming period under transient simulations is selected as an 11-year period centered on the time of 1.5 °C/2.0 °C global warming targets, as shown in Shi *et al*.^15^. The same data manipulation strategy as for HAPPI is used here. That is, relevant diagnoses and indices are calculated at the native grid of each model, and final results are re-gridded to the 1° × 1° grid to facilitate a fair comparison with HAPPI. However, it is to be noted that due to different states in CMIP5 and HAPPI for their own references, they can’t be compared directly. But it is fair and meaningful to compare them when considering the additional 0.5 °C warming from 1.5 to 2.0 °C.

**Table 2:** Basic information on CMIP5 models and the time of 1.5 °C and 2.0 °C global warming.

Model	Resolution (#lat × #long)	Time of 1.5 °C	Time of 2.0 °C
BCC-CSM-1	64 × 128	2023	2045
BCC-CSM1-1-M	160 × 320	2014	2039
CCSM4	192 × 288	2017	2040
CNRM-CM5	128 × 256	2037	2059
CSIRO-MK3-6-0	96 × 192	2035	2048
GFDL-CM3	90 × 144	2023	2037
IPSL-CM5A-LR	96 × 96	2013	2030
IPSL-CM5A-MR	143 × 144	2017	2034
MIROC-ESM-CHEM	64 × 128	2022	2036
MPI-ESM-MR	96 × 192	2023	2045
MPI-ESM-LR	96 × 192	2021	2042
MRI-CGCM3	160 × 320	2054	2085
MIROC5	128 × 256	2040	2072
NorESM1-M	96 × 144	2041	2074
CanESM2	64 × 128	2018	2031

### Methods

#### Extreme indices

We use four extreme temperature indices following the recommendation of the Expert Team on Climate Change Detection and Indices (ETCCDI)^28^, namely annual maximum temperature (TXx), annual minimum temperature (TNn), warm spell duration index (WSDI) and frost days (FD). The definition of these indices is shown in Table 3.

**Table 3:** Definition of 4 ETCCDI temperature indices used in this study.

Abbreviation	Indicator name	Definitions (Units)
TXx	Hottest day	Annual maximum daily maximum temperature (°C)
TNn	Coldest day	Annual minimum daily minimum temperature (°C)
WSDI	Warm spell duration	Annual count of days with at least 6 consecutive days when daily maximum temperature >90th percentile (days)
FD	Frost days	Annual count of days when daily minimum temperature <0 °C (days)

#### Analysis of variance

The analysis of variance is based on variance decomposition which partitions the total observed variance of a given variable into components of separated variation sources. The HAPPI experimental design permits to explore variation of results attributable to inter-model differences and internal (inter-member) variation. This can be served as a surrogate to measure uncertainty of climate projections which is generally considered as from three different sources: emission-scenario uncertainty, model-related uncertainty and internal variability^29^. The HAPPI experiment is obviously not applicable to issues of emission scenarios, but very useful to quantify uncertainties due to models’ spreading and internal variability.

Let *x(m*, *n)* designate a variable (e.g. a mean climate index over China and for a certain duration) obtained from the *m*th one of *M* models (here 4) and the *n*th one of *N* ensemble members (here 100), we can define (similar to Li^30^) two operations of average: mean for each individual model and the multi-model ensemble mean. The mean for each model is:

1 $${x}_{i}(m)=\frac{1}{N}\mathop{\sum }\limits_{n=1}^{N}x(m,n),\,m=1,2,\ldots M.$$

And the multi-model ensemble mean (general mean) is:

2 $${x}_{e}=\frac{1}{M}\mathop{\sum }\limits_{m=1}^{M}{x}_{i}(m).$$

We can demonstrate that the total variance of *x(m*, *n)* is the sum of internal (inter-member) variability ${\sigma }_{N}^{2}$ and models’ spread (inter-model variability) ${\sigma }_{M}^{2}$, calculable as:

3 $${\sigma }_{N}^{2}=\frac{1}{M}\mathop{\sum }\limits_{m=1}^{M}\{\frac{1}{N}\mathop{\sum }\limits_{n=1}^{N}{[x(m,n)-{x}_{i}(m)]}^{2}\}.$$

and

4 $${\sigma }_{M}^{2}=\frac{1}{M}\mathop{\sum }\limits_{m=1}^{M}{[{x}_{i}(m)-{x}_{e}]}^{2}.$$

#### Probability ratio

To assess change of occurrence probabilities of extreme events in the future warmer world, we use the concept of Probability Ratio (PR) between the two probabilities of the event in future (p_1_) and in present day (p_0_):

$${\rm{PR}}={{\rm{p}}}_{1}/{{\rm{p}}}_{0},$$

It represents how much the occurrence probability of a present-day extreme event changes in a future warmer climate^31–33^.

## Results

### Changes in mean and extreme temperature

**Figure 1:**
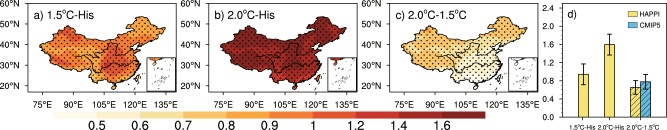
The spatial pattern of annual-mean temperature (**a**–**c**) over China under stabilized 1.5 °C and 2 °C global warming relative to 2006–2015 (Units: °C). Dotted areas are statistically significant according to a two-sample Kolmogorov-Smirnov (K-S) test (p < 0.05). The areal-mean results are shown in Fig. 1d with the yellow bars representing the change of areal-mean temperature of stabilized simulations, and the blue bar representing that of transient simulations; the error bars represent ranges of the mean ± one standard deviation (1*σ*), and the hatching indicates that there is significant difference between transient and stabilized simulations following a K-S test (p < 0.05). The maps were plotted with NCL 6.2.1 (free software; http://www.ncl.ucar.edu/).

Changes of annual-mean temperature over China under 1.5 °C and 2 °C global warming levels are shown in Fig. 1a,b for geographic distributions and in Fig. 1d for the areal-average (grid area-weighted). The areal-mean over China increases by 0.94 °C (0.71–1.17 °C) and 1.59 °C (1.36–1.82 °C) respectively, higher than the global mean which increases by 0.67 °C (0.64–0.7 °C) and 1.15 °C (1.11–1.19 °C) respectively. As generally practiced, changes shown hereafter are relative to present climate (2006–2015), while warming levels (1.5 °C and 2.0 °C) are relative to pre-industrial. Temperature increases over 0.7 °C across China under the level of 1.5 °C global warming. Large values (>1.0 °C) are mainly located in Northwest and Northeast China. They can reach values larger than 1.6 °C under 2.0 °C global warming. We note that the spatial distribution shown here is quite close to what obtained from transient simulations, as shown in previous studies^5,15,34^. For the half-a-degree additional warming, the geographic distribution is shown in Fig. 1c, while the areal average (yellow bars) is shown in Fig. 1d which also adds that from transient simulations (blue bar). Under the additional warming of 0.5 °C, the mean temperature increases mostly in Northwest and Northeast China by more than 0.7 °C. The mean increases are 0.65 °C (0.5–0.8 °C) and 0.78 °C (0.62–0.94 °C) for stabilized and transient simulations respectively. The difference between them is statistically significant according to a two-sample Kolmogorov-Smirnov (K-S) test (p < 0.05). The K-S test is a non-parametric test, suitable for extreme indices which generally show non-Gaussian distribution characteristics^35,36^.

**Figure 2:**
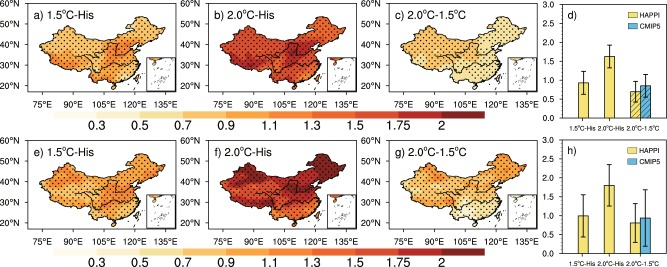
As in Fig. 1, but for TXx (**a**–**d**) and TNn (**e**–**h**) (Units: °C).

Figure 2 depicts changes of TXx and TNn at 1.5 °C and 2 °C warming levels, and in the case of half-a-degree additional warming in panels a, b and c respectively. The areal-mean over China increases by 0.93 °C (0.63–1.23 °C), 1.63 °C (1.33–1.93 °C) for 1.5 °C and 2 °C global warming, and the areal-mean TNn increases by 0.99 °C (0.43–1.55 °C) and 1.8 °C (1.25–2.35 °C) respectively, the magnitude of which is a little larger than TXx, indicating that global warming has more effect on extreme cold events, which is consistent with previous studies^5,37,38^. The largest increase of TXx occurs over North China and the west of the Tibetan Plateau, with 1.1 °C/1.75 °C warmer under 1.5 °C/2.0 °C global warming levels; and TNn increases mostly in northern China, with more than 1.1 °C/2.0 °C warmer in Northwest and Northeast-North China, the spatial distribution in transient simulations also exhibits similarly^15,39^. The magnitudes of both TXx and TNn increase are higher than that for mean temperature in these areas, indicating that extreme temperature events are more sensitive to global warming.

As for the additional warming of 0.5 °C, the areal-mean TXx increases by 0.7 °C (0.43–0.97 °C) in stabilized simulations, while the increase is 0.85 °C (0.55–1.15 °C) in transient simulations (Fig. 2d); and areal-mean TNn increases by 0.81 °C (0.3–1.32 °C) and 0.94 °C (0.19–1.69 °C) respectively (Fig. 2h), it can be seen that the transient response is a little higher than the stabilized one. As shown in Fig. 2c, TXx increases more than 0.5 °C all over China under the additional half-a-degree warming, especially in Northwest China with a higher increase over 0.7 °C; TNn also has an increase of more than 0.5 °C in most regions of China, with the largest increase of more than 0.9 °C in part of Northwest and Northeast China (Fig. 2g).

**Figure 3:**
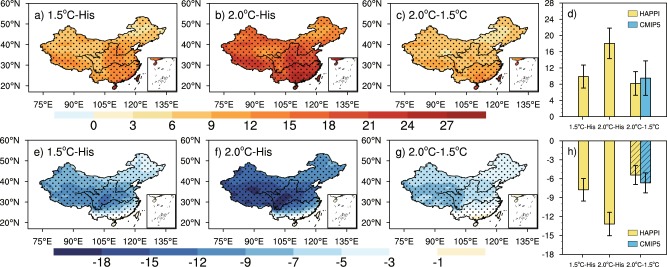
As in Fig. 1, but for WSDI **(a–d)** and FD **(e–h)** (Units: days).

Figure 3 (three columns on the left) shows the projected changes of WSDI and FD under 1.5 °C/2 °C global warming levels and for the half-a-degree warming respectively, while areal-means are shown on the right with results from transient simulations added (blue bar). The areal-mean WSDI increases obviously with global warming, which is 9.9 days (7.1–12.7) and 18.1 days (14.3–21.8) respectively in 1.5 °C and 2.0 °C warmer world. There is an obvious decrease in areal-mean FD, which is 7.8 days (6.0–9.5) and 13.2 days (11.3–15.0), respectively. When considering the additional warming of 0.5 °C, the stabilized response of areal-mean WSDI shows an increase of 8.2 days (5.3–11.2), and the transient response is 9.5 days (5.3–13.8). The decrease of FD is 5.4 days (3.9–6.9) and 6.7 days (5.1–8.2) in the stabilized and transient simulations respectively. For both WSDI and FD, the response in transient simulations is larger than that of stabilized simulations (the last two bars in Fig. 3d,h).

It can be seen that, in terms of spatial distribution, WSDI increases mainly in the south of the Tibetan Plateau and in Southeast China, with an increase reaching 12 days, 21 days and 9 days for the 1.5 °C, 2.0 °C warming levels and the additional warming of 0.5 °C, respectively. These areas would suffer severe heat waves in the future. FD decreases in most parts of China, with the largest decrease in the Tibetan Plateau with values larger than 9 days/15 days under the 1.5 °C/2.0 °C warming levels, and larger than 7 days for the additional 0.5 °C warming, which implies that the Tibetan Plateau would be strongly affected by global warming. The transient response of WSDI and FD based on CMIP5 also exhibits similar response, as shown in Lang *et al*.^40^, Chen *et al*.^37^, Shi *et al*.^15^ and Li *et al*.^41^.

When combining the stabilized response of extreme temperature in China to 1.5 °C/2.0 °C global warming with the results based on transient simulations in previous studies^11,15,41,42^, we can find that the areal-mean extreme temperatures over China show an increase larger than their global counterpart in both stabilized and transient simulations, and key regions are located in Northwest and Northeast-North China; while WSDI increases mostly in Southeast China, FD decreases mostly in the Tibetan Plateau. The results indicate that the response of these regions to global warming is robust, that is, these regions are truly more sensitive to global warming and will suffer severe effects, whether under which warming scenarios. Under the additional warming of 0.5 °C, the change of areal-mean temperature and all extreme indices are larger in transient simulations than that in stabilized simulations (the difference between them is significant in mean temperature, TXx and FD). Moreover, the uncertainty ranges of all areal-mean indices are narrower in stabilized simulations than that in transient simulations, for the ensemble mean of HAPPI project (which has large ensemble members) can reduce the uncertainty caused by climate internal variability^22^. But it should be noted that in this study we only use 4 models of HAPPI project, but 15 models of CMIP5; moreover, the uncertainties of HAPPI results are composed of the spread among different models and different individuals, while the uncertainties of CMIP5 are only due to model spreads.

**Figure 4:**
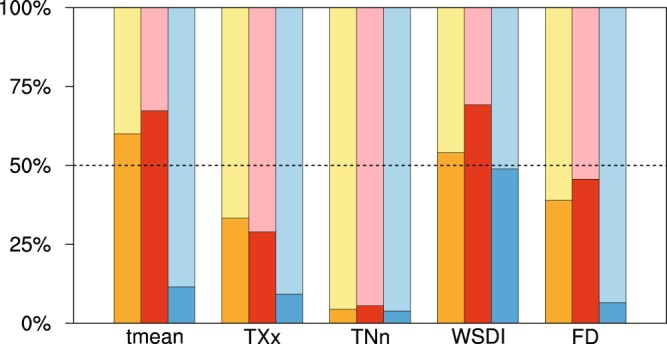
Percentages of variances (for different areal-mean climate indices over China) attributed to inter-model variability (dark colors) and inter-member variability (light colors). The former measures the spreading among the four HAPPI models, the latter measures the internal spreading among the 100 realization members performed within each model. Each group of three bars represents the warming levels 1.5 °C (orange), 2.0 °C (red), and the effect of half-a-degree increase (blue), respectively.

To quantify the two sources of uncertainty in relation to spreading among models and internal variability among different runs, we perform an analysis of variance for the temporal (whole duration of runs) and spatial (whole China) averages of different climate indices. Results are shown in Fig. 4. It can be seen that, under 1.5 °C (dark orange and light yellow) and 2.0 °C (dark red and light red) global warming, the mean temperature shows a larger inter-model spreading (60 and 67%) against internal variability. For most extreme indices, we observe a dominant internal variability, except for WSDI which shows a large spreading among models (consistent with what found in Shi *et al*.^15^ for models running transient scenarios). For an additional warming of 0.5 °C (blue) and for all indices, the variance is mainly brought in by internal variability whose contribution is more than 85%. WSDI again shows a particular behavior with an important inter-model spreading.

### Changes in occurrence probabilities for hot extremes

**Figure 5:**
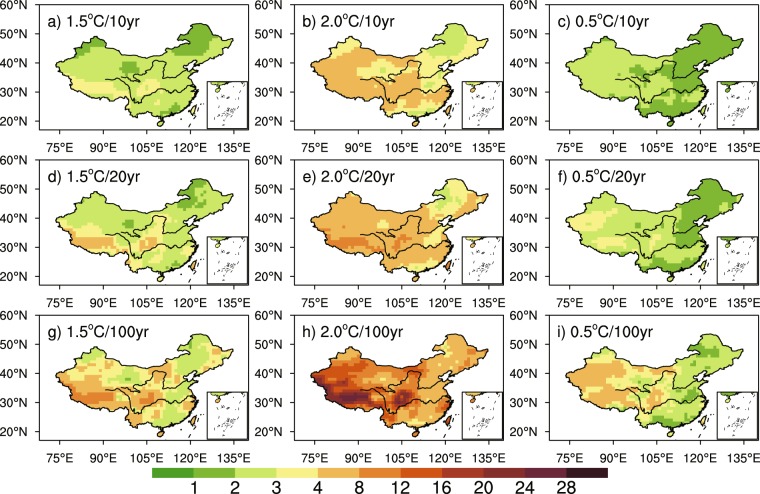
The spatial patterns of PR for 10-year (**a**–**c**), 20-year (**d**–**f**) and 100-year TXx (**g**–**i**) under 1.5 °C (first column) and 2.0 °C (second column) global warming and the additional warming of 0.5 °C (third column) based on stabilized simulations. The maps were plotted with NCL 6.2.1 (free software; http://www.ncl.ucar.edu/).

We firstly examine the spatial distributions of PR for extreme hot events calculated at each grid point. We used Generalized Extreme Value (GEV) approach to evaluate their probability and PR from the reference period to warming scenario. The methodology that we used is the same as in Kharin *et al*.^43^. It is to be noted that important biases may exist among various models and their individual members^19^, which makes the ensemble of data incoherent among them and inapplicable for a GEV distribution. To remediate this issue, we use anomalous fields calculated from each individual model, and add the general mean value just before performing the GEV fitting. After the calibration of the three free parameters of GEV (location, scale, and shape), the PR is calculated for three probabilities, 90%, 95% and 99%, respectively, corresponding to events that occur every 10, 20 and 100 years. The PR of extreme hot events increases over China with global warming (Fig. 5). It can be seen in Fig. 5a–h that the spatial patterns of PR are similar between 1.5 °C and 2.0 °C global warming, whereas the value is larger for a higher warming level and a rarer event. The PR is relatively high in the south of the Tibetan Plateau and Northwest China in a future warmer world, with occurrence probability for 100-year extreme hot event increases by 4 and 3 times in these regions respectively (that is, the return values of such event change to 25.0 and 33.3 years) under 1.5 °C global warming; and in a 2.0 °C warmer world, the occurrence probability for 100-year extreme hot event becomes 16 and 8 times respectively (the return values of 100-year extreme hot event change to 6.3 and 12.5 years). The additional warming of 0.5 °C leads to higher PR in Northwest China and the occurrence probability for 100-year extreme hot event increases by about 4 times (the return value is 25.0 years) relative to the 1.5 °C warming level.

Results shown in Fig. 5 can also be summarized in box-whisker plots as in Supplementary Fig. S1 to highlight the characteristics of PR spatial distribution in China. Under 1.5 °C warming, the median PR for 10-, 20- and 100-year TXx over China is 2.35, 2.69 and 3.68, and the range of which is 1.55–3.8, 1.64–5.06 and 1.81–11.42 respectively, with the biggest PR located in the south of the Tibetan Plateau. The values and ranges of PR are both larger under 2.0 °C global warming, indicating that the occurrence probabilities of extreme events will change a lot and the difference between regions will become larger when global warming rises from 1.5 °C to 2.0 °C.

We now investigate how extreme events would change at the national level. To do so, we make the areal average, over whole China, of TXx for each year of our considered periods. We are aware that averaging over large domain for extreme indices (such as TXx) may not have a physical significance, since they may be geographically independent for different grids. However, they may have a practical meaning for policy decision makers to have a national-level evaluation of extreme events. This gives us an ensemble of areal-mean values which forms a probability distribution. Despite the averaging operation over space, such a distribution does not pass the normality test. A GEV distribution is then used to fit the data.

**Figure 6:**
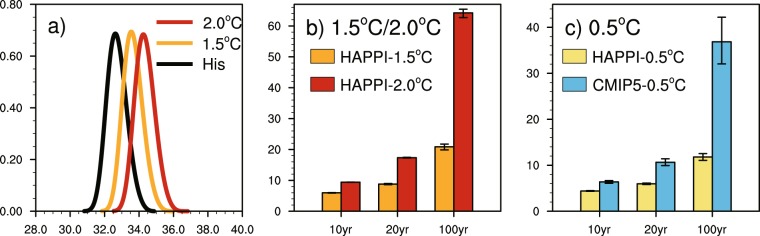
The PDF of areal-mean TXx series over China under stabilized 1.5 °C and 2.0 °C global warming (**a**) and the PR for 10-, 20- and 100-year areal-mean TXx in stabilized and transient simulations (**b**, **c**). The orange/red bars represent the PR under 1.5 °C/2.0 °C stabilized warming (**b**), while the yellow/blue bars represent the PR under the additional 0.5 °C warming in stabilized/transient simulations (**c**). The median (bars in **b**,**c**) and 25–75% uncertainty range (vertical lines in **b**,**c**) derived from 1000 bootstrapped subsamples are shown.

The PDF curves and PR for areal-mean TXx are shown in Fig. 6, the uncertainty ranges of PR for areal-mean TXx series are estimated from 1000 bootstrapped subsamples. As shown in Fig. 6a, The PDF curves of TXx shift toward right, indicating an increase of TXx with warming; and the shape of PDF curves doesn’t change too much, meaning that there is no obvious change in variability of TXx. Under 1.5 °C global warming, the occurrence probabilities for present 10-, 20- and 100-year TXx increase by 5.96 times (5.88–6.04), 8.79 times (8.61–8.98) and 20.86 times (19.86–21.75) respectively, while under 2.0 °C warming the increase up to 9.42 times (9.39–9.45), 17.34 times (17.23–17.45) and 64.24 times (62.71–65.43) as in Fig. 6b. The rarest extreme events receive the largest value of PR, especially for the higher global warming. The extreme hot event with 100-year return period is expected to occur every 4.79 years in a 1.5 °C warmer world and every 1.56 years in a 2.0 °C warmer world, the quite large difference between the return periods under different warming levels suggests that the probability for severely extreme hot event will experience an intensive increase in higher global warming scenarios.

Figure 6c shows PR for the additional 0.5 °C warming. Also shown is the counterpart from transient simulations. The occurrence probability for 10-, 20- and 100-year TXx increases by 4.40 times (4.34–4.48), 5.97 times (5.82–6.13) and 11.79 times (11.07–12.53) in stabilized simulations, respectively, while it increases by 6.38 times (6.07–6.67), 10.65 times (9.92–11.40) and 36.86 times (32.04–42.19) in transient simulations. The two modes of running simulations both suggest that limiting global warming at 1.5 °C can effectively reduce probabilities for extreme hot events. The occurrence probabilities for extreme hot events with different return periods in transient simulations are higher than that in stabilized simulations under the additional 0.5 °C warming. And the uncertainty ranges of projected occurrence probabilities for extreme hot events are obviously narrower in stabilized simulations than in transient simulations, due to the large ensemble members of HAPPI data.

### Changes in occurrence probabilities for cold extremes

**Figure 7:**
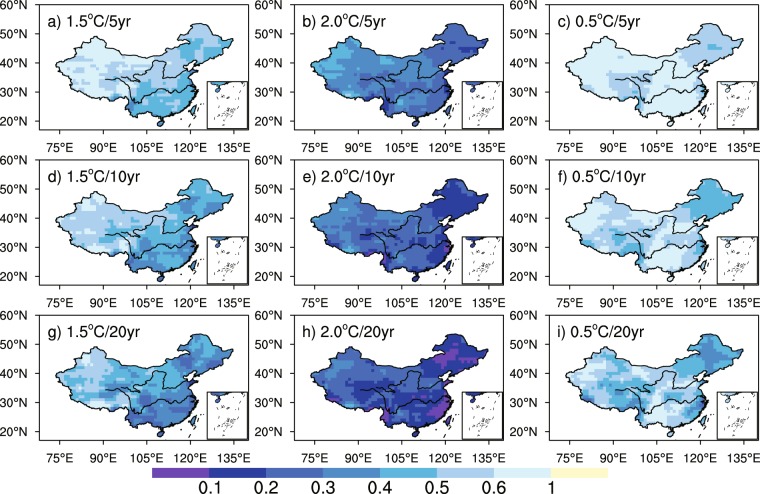
As in Fig. 5, but for 5-, 10- and 20-year TNn.

Figure 7 shows the spatial patterns of PR for extreme cold events in stabilized simulations. The GEV estimation is also used to evaluate the occurrence probability of TNn. Note that GEV distribution is only suitable for extreme large values, thus we simply take a negative sign for TNn when calculating PR. Considering the probability of 100-year TNn is too small and contains large uncertainty, we analyze the extreme cold event with 5-, 10-, and 20-year return periods. And we also remove the systematic biases among models in TNn series before further processing, as what we did to TXx series in Fig. 5. There are very similar spatial patterns between the 1.5 °C and 2.0 °C warming targets, whereas rarer events have much smaller values, especially over higher warming levels (Fig. 7a–h). For an extreme cold event in present climate, a smaller PR means that it has a lower probability to occur under future warming scenarios. The occurrence probabilities of extreme cold events reduce all over China (PR < 1) in warmer worlds, areas with smaller PR located in the east of the Tibetan Plateau, Northeast and Southeast China, the PR of 10-year TNn in the present climate is 0.5/0.3 under 1.5 °C/2.0 °C global warming, that is, the return period of which is 20/33 years. Under the additional 0.5 °C warming, the PR of 10-year TNn is still relatively small in the east of the Tibetan Plateau and Northeast China (PR is 0.5 approximately), indicating that a TNn event expected once every 10 years in a 1.5 °C warmer world is expected to occur about every 20 years in a 2.0 °C warming climate, suggesting that limiting global warming at 1.5 °C will reduce the change of occurrence probabilities for extreme cold events in these regions, which is also identified in Supplementary Fig. S2. It can be seen in Fig. S2 that the median PR for 5-, 10- and 20-year TNn over China is 0.54, 0.48 and 0.42 under 1.5 °C warming, and the range of which is 0.31–0.75, 0.2–0.76 and 0.11–0.8 respectively. The PR values are much lower in a 2.0 °C warmer world, with the minimum PR for 20-year TNn closed to 0 (located in Southeast China), meaning that the once-in-20-year TNn happened in present climate may not occur in this area under 2.0 °C global warming.

To understand how extreme cold events would change at the national level, the PR and PDF for areal-mean TNn series are shown in Supplementary Fig. S3. As in Fig. S3a, the right shift of the PDF indicates an increase of TNn with global warming, which also suggests that the occurrence probabilities of present extreme cold events may become very small in the warmer worlds. The shape of PDF curves doesn’t change much, meaning that there are no obvious changes in variability of TNn under stabilized simulations. When global warming stabilized at 1.5 °C, the PR for 5-, 10- and 20-year TNn is 0.23 (0.21–0.24), 0.15 (0.14–0.17) and 0.10 (0.08–0.11), respectively; and in a 2.0 °C warmer world the PR for TNn with different return levels is 0.039 (0.034–0.044), 0.013 (0.010–0.018) and 0.003 (0.001–0.006) respectively (Supplementary Fig. S3b). It can be seen that the values of PR for TNn will be smaller with higher global warming levels and rarer extreme cold events. For example, the areal-mean extreme cold event expected every 10 years in the present climate is expected about every 67 years under stabilized 1.5 °C global warming, while under 2.0 °C warming the return period of which will be more than 700 years, meaning that such extreme cold event is unlikely to happen in a 2.0 °C warmer world.

For the additional 0.5 °C warming, the PR for 5-, 10- and 20-year TNn is 0.33 (0.32–0.34), 0.25 (0.23–0.67) and 0.2 (0.16–0.21) respectively relative to that under stabilized 1.5 °C global warming; while in transient simulations, the PR for TNn with different return levels is 0.32 (0.26–0.38), 0.29 (0.21–0.37) and 0.29 (0.18–0.42) respectively (Supplementary Fig. S3c), the results between different return periods are similar and hold wider uncertainty ranges than stabilized results, due to the quite small ensemble members compared to HAPPI simulations. Both of the warming simulations indicate that occurrence probabilities for extreme cold events will be mitigated when limiting global warming at 1.5 °C.

## Conclusions

In this study we used HAPPI datasets to analyze the response of extreme temperature in China when global warming stabilizes at 1.5 °C/2.0 °C, and compared the response difference between stabilized and transient simulations under the additional 0.5 °C warming. Main results are as follows:

1. Under stabilized 1.5 °C/2.0 °C global warming (relative to pre-industrial), the global mean temperature increases by 0.67 °C/1.15 °C relative to present climate (2006–2015), and the increase of areal-mean temperature over China is higher, which is 0.94 °C/1.59 °C; TXx increases by 0.93 °C/1.63 °C, TNn increases by 0.99 °C/1.8 °C, WSDI increases by 9.9 days/18.1 days and FD decreases by 7.8 days/13.2 days. Under the additional warming of 0.5 °C, the change of mean and extreme temperature is higher in transient simulations (multi-model ensemble from CMIP5 datasets) than that in stabilized simulations. The uncertainty ranges are narrowed in stabilized simulations, due to the large ensemble members in HAPPI datasets.
2. Under stabilized warming scenarios, the mean temperature, TXx and TNn increase mostly in Northwest and Northeast-North China, the increase is about 1.1 °C in 1.5 °C warmer world, and is higher in 2.0 °C. TXx and TNn have a higher increase compared to the mean temperature; WSDI increases mostly in Southeast China, which increases more than 12 days/21 days under 1.5 °C/2.0 °C global warming; and FD decreases more than 9 days/15 days in the Tibetan Plateau. The results are similar with those from transient simulations, indicating that these areas are sensitive to global warming in both stabilized and transient warming scenarios.
3. The HAPPI experimental design, with a large ensemble size, allowed us to decompose the total variance into a part related to inter-model variability and another related to internal variability. This decomposition serves as a surrogate to estimate uncertainties of climate projections. In the case of 1.5 °C and 2.0 °C warming levels, the two variances are roughly equal (about 50% each) for the mean temperature, WSDI and FD, while the internal variability dominates for TXx and TNn. In the case of additional half-a-degree warming, the internal variability is clearly dominant over the inter-model variability (80 versus 20%) for most variables (except for WSDI which shows half-and-half).
4. The PR of extreme hot/cold events will increase/decrease under global warming. There is larger change of occurrence probabilities in higher global warming levels, especially for rarer extreme events. For geographic distribution, the occurrence probabilities for TXx are higher in the south of the Tibetan Plateau, where the return period of 100-year TXx changes to 25/6.3 years under stabilized 1.5 °C/2.0 °C global warming; the occurrence probabilities for TNn change mostly in the east of the Tibetan Plateau, Northeast and Southeast China, where the 10-year TNn event becomes 20/33 years, which can be inferred that limiting global warming at 1.5 °C can reduce the change of occurrence probabilities for extreme temperature events. For the national averaged extreme events, the return period of 100-year TXx over China changes to 4.79 and 1.56 years under stabilized 1.5 °C and 2.0 °C global warming, and the return period of 10-year TNn changes to 67 years and more than 700 years; for the additional warming of 0.5 °C, the uncertainty ranges of PR for areal-mean TXx and TNn are obviously narrower in stabilized simulations than that in transient simulations.

## Supplementary information

Supplementary Information

## Data Availability

The datasets generated and analyzed during the current study are available from the corresponding author on request.

## References

[1] Intergovernmental Panel on climate Change (IPCC). Climate Change 2013: *The Physical Science Basis. Contribution of Working Group I to the Fifth Assessment Report of the Intergovernmental Panel on Climate Change*. Stocker, T. F. *et al*., Eds, www.ipcc.ch/report/ar5/wg1 (IPCC, World Meteorological Organization, United Nations Environment Programme, Berlin, Germany, 2013).

[2] UNFCC, Adoption of the Paris Agreement. Report No. FCCC/CP/2015/L.9/Rev.1, http://unfccc.int/resource/docs/2015/cop21/eng/l09r01.pdf, *United Nations Framework Convention on Climate Change* (2015).

[3] Intergovernmental Panel on climate Change (IPCC), “Summary for policymakers: Global warming of 1.5 °C—An IPCC Special Report on the impacts of global warming of 1.5 °C above pre-industrial levels and related global greenhouse gas emission pathways, in the context of strengthening the global response to the threat of climate change, sustainable development, and eforts to eradicate poverty”, V. Masson-Delmotte *et al*., Eds, www.ipcc.ch/report/sr15 (IPCC, World Meteorological Organization, United Nations Environment Programme, Geneva, Switzerland, 2018).

[4] Knutti R, Rogelj J, Sedláček J, Fischer EM (2015). A scientific critique of the two-degree climate change target. Nat. Geosci..

[5] Hu T, Sun Y, Zhang X (2017). Temperature and precipitation projection at 1.5 and 2 °C increase in global mean temperature. Chin. Sci. Bull..

[6] Xu Y (2017). Asian climate change under 1.5–4 °C warming targets. Adv. Climate Change Res..

[7] Karmalkar AV, Bradley RS (2017). Consequences of global warming of 1.5 °C and 2 °C for regional temperature and precipitation changes in the contiguous United States. PLoS One.

[8] Park CE (2018). Keeping global warming within 1.5 °C constrains emergence of aridification. Nat. Clim. Chang..

[9] King AD, Karoly DJ, Henley BJ (2017). Australian climate extremes at 1.5 °C and 2 °C of global warming. Nat. Clim. Chang..

[10] Wartenburger R (2017). Changes in regional climate extremes as a function of global mean temperature: an interactive plotting framework. Geosci. Model Dev..

[11] Chen H, Sun J (2018). Projected changes in climate extremes in China in a 1.5 °C warmer world. Int. J. Climatol..

[12] Dosio A, Fischer EM (2018). Will half a degree make a difference? Robust projections of indices of mean and extreme climate in Europe under 1.5 °C, 2 °C, and 3 °C global warming. Geophys. Res. Lett..

[13] Schleussner C-F (2016). Differential climate impacts for policy-relevant limits to global warming: the case of 1.5 °C and 2 °C. Earth. Syst. Dynam..

[14] Wang Z (2017). Scenario dependence of future changes in climate extremes under 1.5 °C and 2 °C global warming. Sci. Rep..

[15] Shi C, Jiang Z, Chen W, Li L (2018). Changes in temperature extremes over China under 1.5 °C and 2 °C global warming targets. Adv. Climate Change Res..

[16] Su B (2018). Drought losses in China might double between the 1.5 °C and 2.0 °C warming. Proc. Natl. Acad. Sci. USA.

[17] Dosio A, Mentaschi L, Fischer EM, Wyser K (2018). Extreme heat waves under 1.5 °C and 2 °C global warming. Environ. Res. Lett..

[18] Zhan M (2018). Changes in extreme maximum temperature events and population exposure in China under global warming scenarios of 1.5 and 2.0 °C: analysis using the regional climate model COSMO-CLM. J. Meteorol. Res..

[19] Mitchell D (2017). Half a degree additional warming, prognosis and projected impacts (HAPPI): background and experimental design. Geosci. Model Dev..

[20] James R, Washington R, Schleussner CF, Rogelj J, Conway D (2017). Characterizing half-a-degree difference: a review of methods for identifying regional climate responses to global warming targets. Wiley Interdiscip. Rev.-Clim. Chang..

[21] Sanderson BM (2017). Community climate simulations to assess avoided impacts in 1.5 and 2 °C futures. Earth Syst. Dynam..

[22] Li C (2018). Midlatitude atmospheric circulation responses under 1.5 and 2.0 °C warming and implications for regional impacts. Earth Syst. Dynam..

[23] Wehner M (2018). Changes in extremely hot days under stabilized 1.5 and 2.0 °C global warming scenarios as simulated by the HAPPI multi-model ensemble. Earth Syst. Dynam..

[24] Chevuturi, A., Klingaman, N. P., Turner, A. G. & Hannah, S. Projected changes in the Asian-Australian monsoon region in 1.5 °C and 2.0 °C global-warming scenarios. *Earth Future*, **6**, 10.1002/2017EF000734 (2018).

[25] Russo S (2019). Half a degree and rapid socioeconomic development matter for heatwave risk. Nat. Commun..

[26] Harrington LJ, Otto FEL (2018). Changing population dynamics and uneven temperature emergence combine to exacerbate regional exposure to heat extremes under 1.5 °C and 2 °C of warming. Environ. Res. Lett..

[27] Lewis SC, King AD, Mitchell DM (2017). Australia’s unprecedented future temperature extremes under Paris Limits to warming. Geophys. Res. Lett..

[28] Zhang X (2011). Indices for monitoring changes in extremes based on daily temperature and precipitation data. Wiley Interdiscip. Rev.-Clim. Chang..

[29] Hawkins E, Sutton R (2009). The potential to narrow uncertainty in regional climate predictions. Bull. Am. Meterol. Soc..

[30] Li ZX (1999). Ensemble atmospheric GCM simulation of climate interannual variability from 1979 to 1994. J. Clim..

[31] Stott PA, Stone DA, Allen MR (2004). Human contribution to the European heatwave of 2003. Nature..

[32] Fischer EM, Knutti R (2015). Anthropogenic contribution to global occurrence of heavy-precipitation and high-temperature extremes. Nat. Clim. Chang..

[33] Stott PA (2016). Attribution of extreme weather and climate-related events. Wiley Interdiscip. Rev.-Clim. Chang..

[34] Zhou T (2018). When and how will the Millennium Silk Road witness 1.5 °C and 2 °C warmer worlds. Atmos. Oceanic Sci Lett..

[35] Sun Q, Miao C, AghaKouchak A, Duan D (2017). Unraveling anthropogenic influence on the changing risk of heat waves in China. Geophys. Res. Lett..

[36] Qian C, Zhang X, Li Z (2019). Linear trends in temperature extremes in China, with an emphasis on non-Gaussian and serially dependent characteristics. Clim. Dynam..

[37] Chen H, Sun J (2015). Changes in climate extreme events in China associated with warming. Int. J. Climatol..

[38] Seneviratne SI (2018). Climate extremes, land– climate feedbacks and land-use forcing at 1.5 °C. Phil.Trans.R. Soc. A.

[39] Zhai, P. *et al*. Research progress in impact of 1.5 °C global warming on global and regional scales. *Adv. Climate Change Res*, **13**, 465–472 (2017). (in Chinese).

[40] Lang, X. & Sui, Y. Changes in mean and extreme climates over China with a 2 °C global warming. Chin. *Sci. Bull*. **58**, 1453–1461 (2013). (in Chinese).

[41] Li D, Zou L, Zhou T (2018). Extreme climate event changes in China in the 1.5 and 2 °C warmer climates: results from statistical and dynamical downscaling. J. Geophys. Res.-Atmos..

[42] Guo X, Huang J, Luo Y, Zhao Z, Xu Y (2016). Projection of heat waves over China for eight different global warming targets using 12 CMIP5 models. Theor. Appl. Climatol..

[43] Kharin VV, Zwiers FW, Zhang X, Wehner M (2013). Changes in temperature and precipitation extremes in the CMIP5 ensemble. Clim. Change..

