# Supplementary material for: Changes in extreme temperature over China when global warming stabilized at 1.5 °C and 2.0 °C
Source: Sci Rep. 2019 Oct 18;9:14982. doi: 10.1038/s41598-019-50036-z (PMC6800422; doi:10.1038/s41598-019-50036-z)
Supplement: Supplementary file 1 — Supplementary Information [file 41598_2019_50036_MOESM1_ESM.pdf]

Supplementary Information for ‘Changes in extreme temperature over China when global warming stabilized at 1.5 °C and 2.0 °C’

Cenxiao Sun[1], Zhihong Jiang[1], Wei Li[1], Qiyao Hou[1]&Laurent Li[2]

[1] Key Laboratory of Meteorological Disaster of Ministry of Education, Collaborative Innovation Center on Forecast and Evaluation of Meteorological Disasters, Nanjing University of Information Science and Technology, Nanjing, 210044, China

[2] Laboratoire de Météorologie Dynamique, IPSL, CNRS, Sorbonne Université, Ecole Normale Supérieure, Ecole Polytechnique, Paris, France

Correspondence and requests for materials should be addressed to Z.J. (email: zhjiang@nuist.edu.cn)

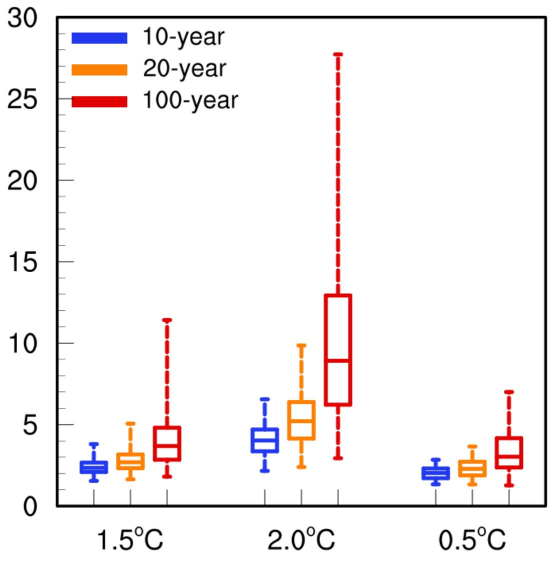

Figure S1. Boxplot of PR for 10-year (blue boxes), 20-year (orange boxes) and 100-year TXx (red boxes) under 1.5°C, 2.0°C and the additional 0.5°C global warming based on stabilized simulations. The top whiskers, top boxes, the bands inside the boxes, bottom boxes and bottom whiskers represent the maximum, 75%, medium, 25% and minimum PR values for TXx in one grid of Figure 5, respectively.

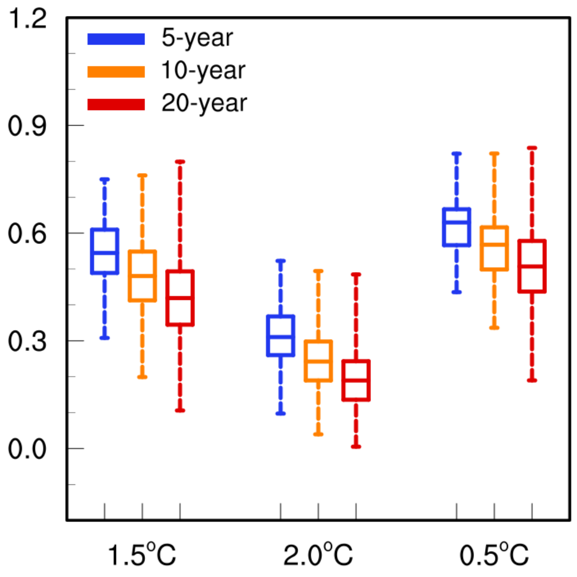

Figure S2. As in Fig. S1, but for 5-, 10- and 20-year TNn.

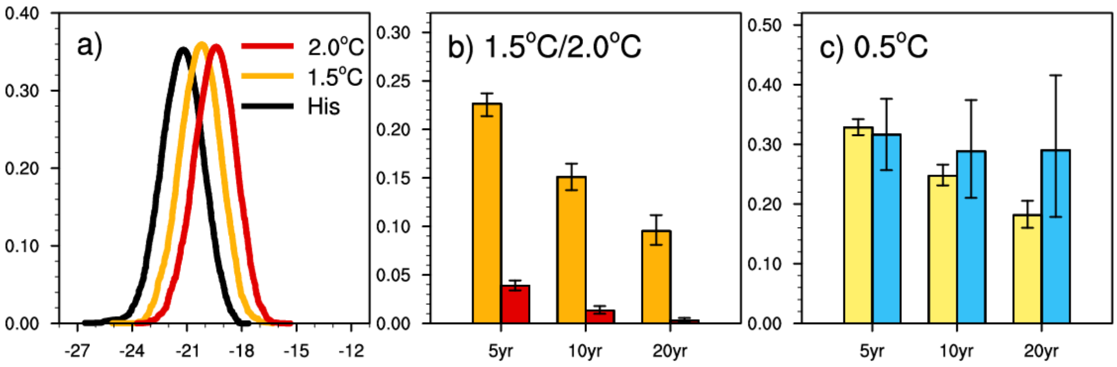

Figure S3. As in Fig. 6, but for 5-, 10- and 20-year TNn.
